# Apparent Endoscopic Decrease in the Size of a Large Ulcerated Gastric Schwannoma After *Helicobacter pylori* Eradication Therapy: A Case Report

**DOI:** 10.1002/deo2.70416

**Published:** 2026-08-26

**Authors:** Takahito Awatsu, Daisuke Asaoka, Hirofumi Fukushima, Daishi Kabemura, Yusuke Nomoto, Osamu Nomura, Kazunori Kajino, Mariko Hojo, Akihito Nagahara

**Affiliations:** ^1^ Department of Gastroenterology Juntendo University Faculty of Medicine Tokyo Japan; ^2^ Department of Gastroenterology Juntendo Tokyo Koto Geriatric Medical Center Tokyo Japan; ^3^ Department of Diagnostic Pathology Juntendo Tokyo Koto Geriatric Medical Center Tokyo Japan

**Keywords:** eradication, gastric schwannoma, gastric ulcer, *Helicobacter pylori*, subepithelial lesion (SEL)

## Abstract

Gastric subepithelial lesions (SELs) comprise a heterogeneous group of tumors, among which gastric schwannomas are rare and generally benign. We report a case of a large gastric schwannoma with ulceration and symptomatic anemia that showed an apparent endoscopic decrease in size following *Helicobacter pylori* eradication.

An 85‐year‐old man presented with iron deficiency anemia. Esophagogastroduodenoscopy revealed a large ulcerated SEL in the lower body of the stomach, along with multiple gastric ulcers. Endoscopic ultrasonography demonstrated a 56 mm tumor originating from the fourth layer, raising suspicion for a gastrointestinal stromal tumor. Boring biopsy from the ulcer base enabled a definitive diagnosis of gastric schwannoma based on immunohistochemical findings. Although surgical resection was recommended, the patient declined. After successful *H. pylori* eradication, follow‐up endoscopy at 6 months showed healing of the ulcerative lesion and an apparent endoscopic decrease in the size of the tumor. Although objective post‐treatment assessment was unavailable, this case suggests that inflammatory or ulcer‐related changes associated with *H. pylori* infection may influence the endoscopic appearance of gastric SELs.

## 1 Introduction

Gastric subepithelial lesions (SELs) comprise a heterogeneous group of tumors, including gastrointestinal stromal tumors, leiomyomas, and schwannomas [1, 2]. Among these, gastric schwannomas are rare mesenchymal tumors, accounting for approximately 0.2% of all gastric neoplasms, and are generally considered benign [3]. Their clinical presentation varies depending on tumor size, and larger lesions may present with symptoms such as bleeding or anemia due to mucosal ulceration. Although most gastric schwannomas follow a benign course, large size and ulcer formation may make it difficult to distinguish them from potentially malignant SELs, particularly gastrointestinal stromal tumors [3].

In contrast, infection with *Helicobacter pylori* is a well‐established cause of chronic gastritis and peptic ulcer disease, leading to mucosal inflammation and ulcer formation [4, 5]. Eradication therapy results in mucosal healing and resolution of inflammation; however, its influence on the morphology or apparent size of SELs remains unclear.

Herein, we report a case of a large gastric schwannoma with ulceration and symptomatic anemia that showed an apparent endoscopic decrease following *H. pylori* eradication therapy, suggesting that inflammatory changes may influence the endoscopic appearance of gastric SELs.

## 2 Case Report

An 85‐year‐old man presented with exertional dyspnea. Laboratory examination revealed iron deficiency anemia (hemoglobin, 6.8 g/dL; serum iron, 11 µg/dL; ferritin, 6.0 ng/mL).

**FIGURE 1:**
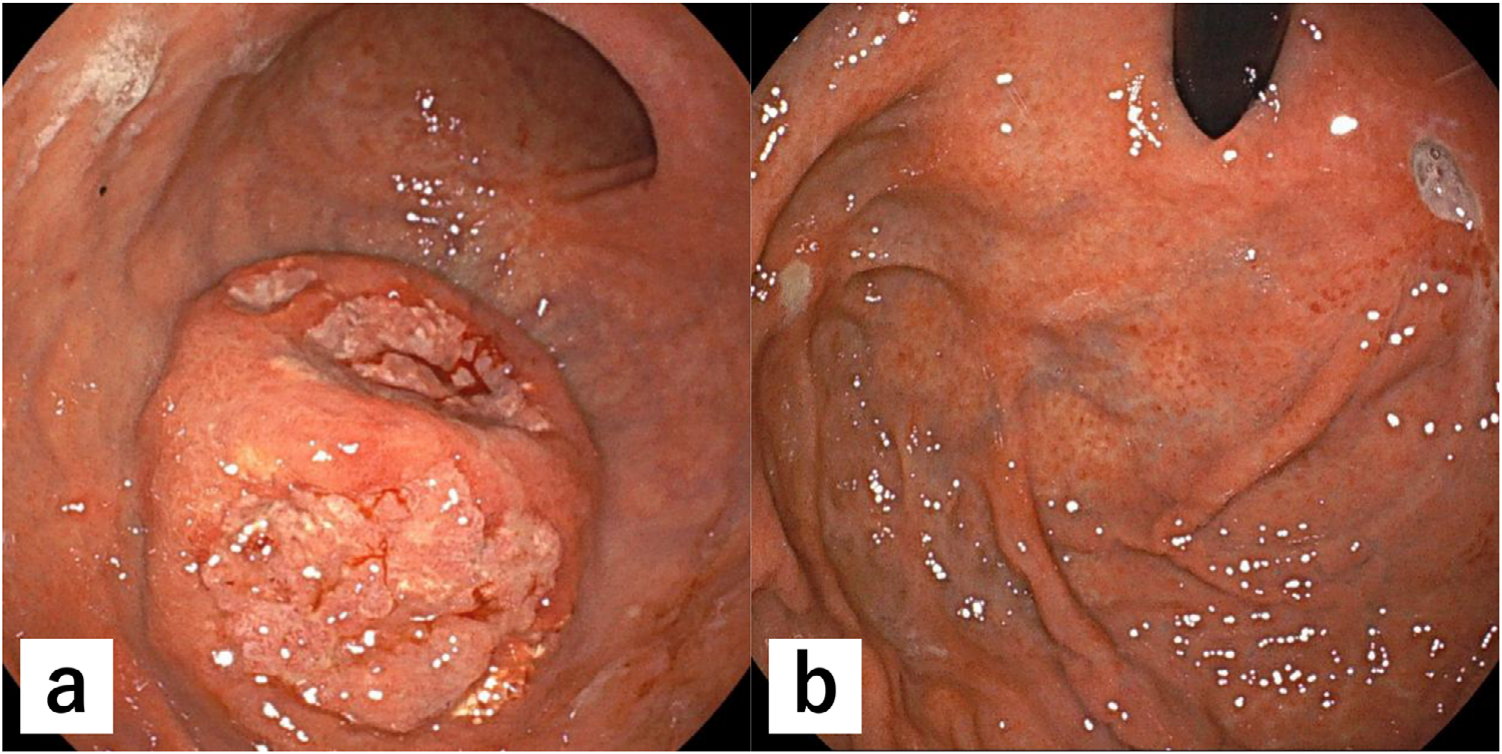
Endoscopic findings. (a) A large gastric subepithelial lesion with ulceration located on the greater curvature of the lower gastric body. (b) Multiple gastric ulcers were observed.

Esophagogastroduodenoscopy (EGD) performed to investigate the cause of anemia revealed a large gastric SEL with ulceration located on the greater curvature of the lower body of the stomach (Figure 1a). Blood was slowly oozing from the ulcer on the SEL. In addition, multiple gastric ulcers were observed (Figure 1b). Biopsy specimens obtained from the ulcer margin of the SEL showed only inflammatory changes, with no evidence of neoplastic cells. Biopsies from the other gastric ulcers revealed no malignant findings, and *H. pylori* organisms were identified histologically.

**FIGURE 2:**
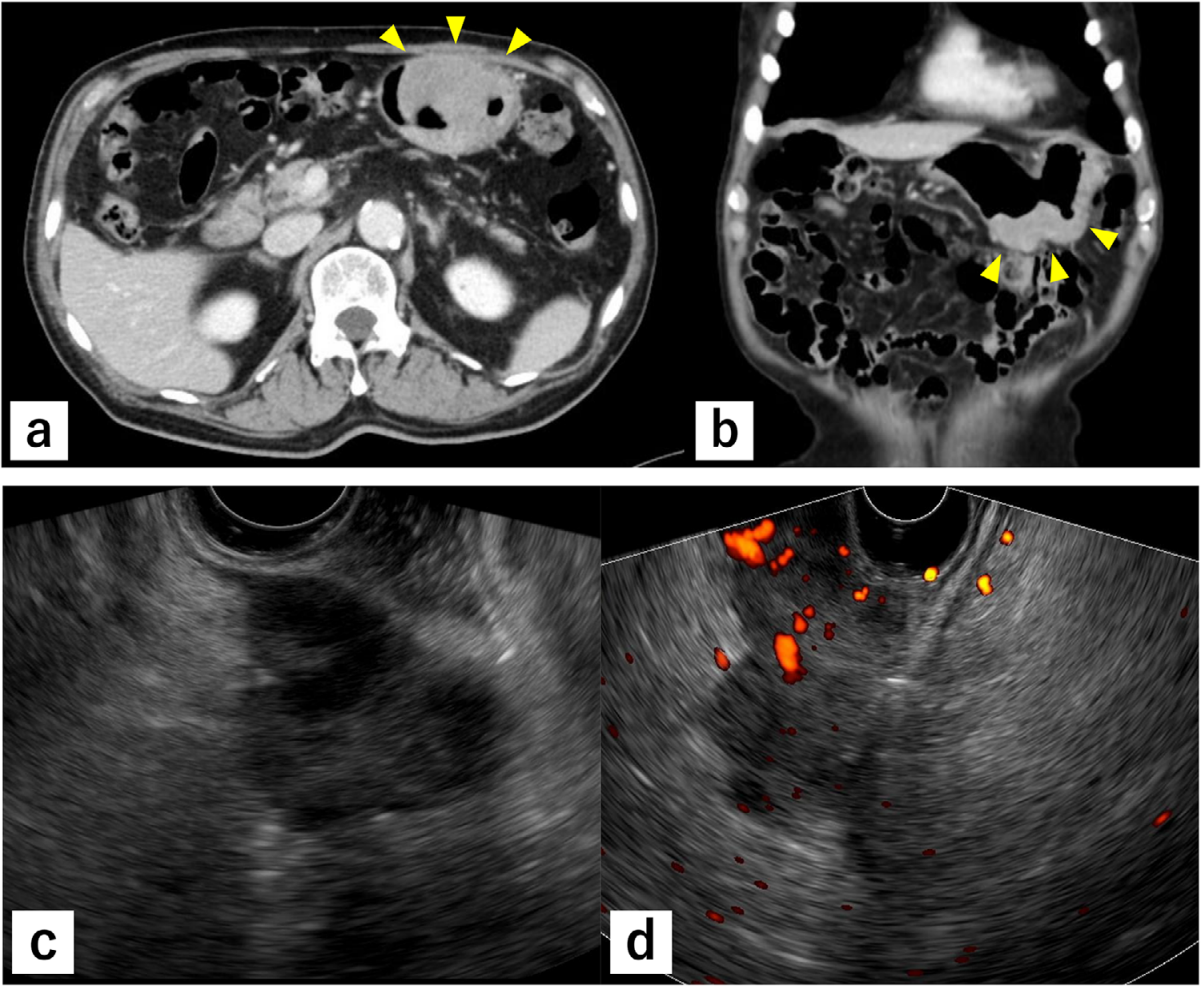
(a, b) Contrast‐enhanced computed tomography demonstrated a well‐enhanced, homogeneous gastric mass without significant lymphadenopathy or distant metastasis. (c) Endoscopic ultrasonography (EUS) revealed a 56‐mm lesion originating from the fourth layer of the gastric wall with heterogeneous echogenicity and partially anechoic areas. (d) Doppler imaging showed internal vascularity within the tumor.

Colonoscopy showed no abnormalities that could explain the anemia. Contrast‐enhanced computed tomography demonstrated a well‐enhanced, homogeneous mass without significant lymphadenopathy or evidence of distant metastasis (Figure 2a,b). Endoscopic ultrasonography (EUS) revealed a 56 mm lesion originating from the fourth layer of the gastric wall (Figure 2c). The tumor exhibited heterogeneous echogenicity with partially anechoic areas and showed internal vascularity on Doppler imaging (Figure 2d). Based on the ulcerated endoscopic appearance and EUS findings, gastrointestinal stromal tumor was considered an important differential diagnosis.

**FIGURE 3:**
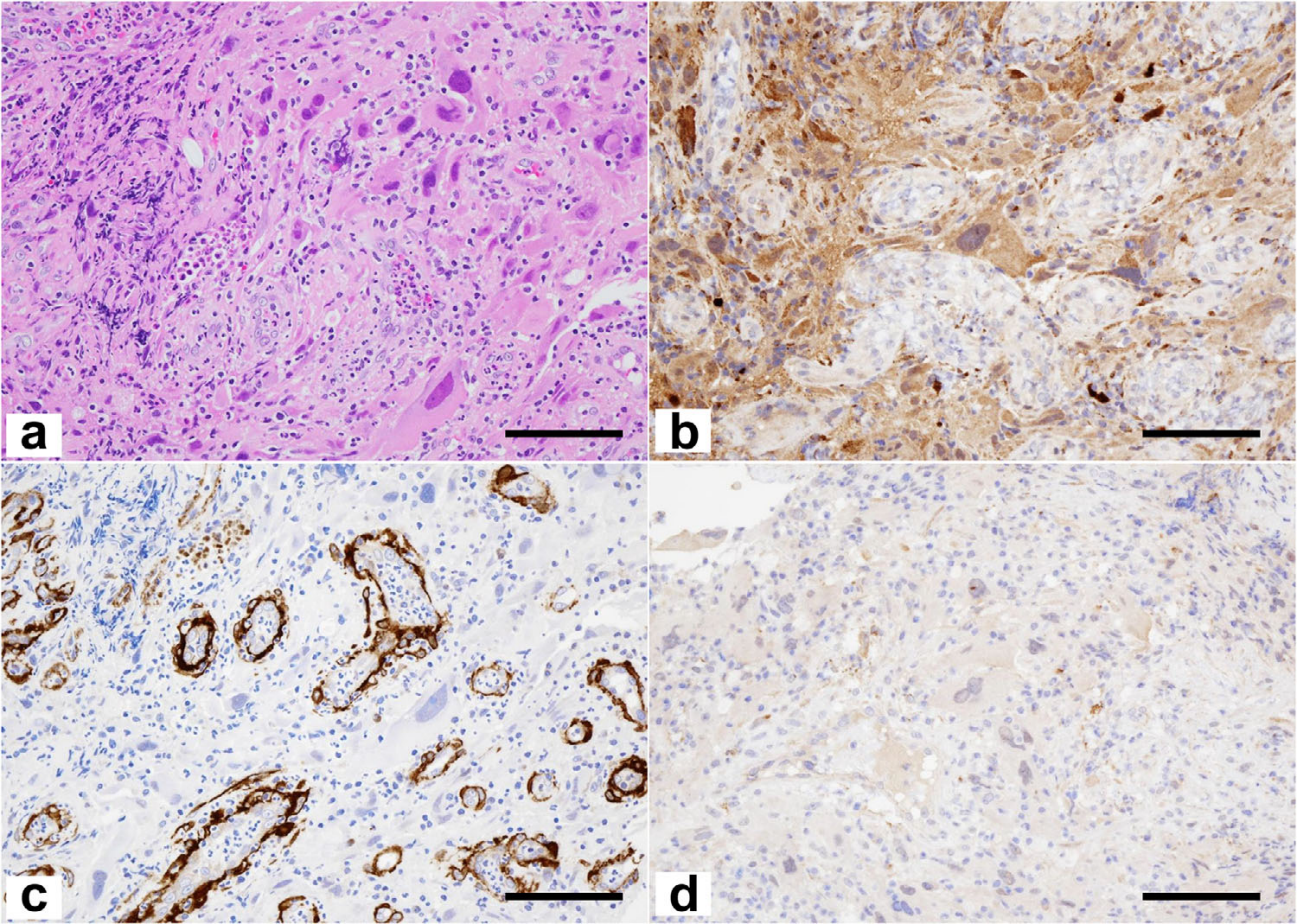
Histopathological and immunohistochemical features of the biopsy specimen. Scale bar: 100 µm. (a) Hematoxylin and eosin staining showed proliferation of spindle‐shaped to oval tumor cells with moderate to severe inflammatory cell infiltration, including plasma cells and neutrophils. (b) Immunohistochemical staining for S‐100 protein showed diffuse positivity in tumor cells. (c) α‐SMA staining was negative. (d) c‐kit staining was negative.

As tumor tissue was macroscopically exposed at the ulcer base, a boring biopsy was performed. Histopathological examination revealed proliferation of spindle‐shaped to oval cells, accompanied by moderate to severe inflammatory cell infiltration, including plasma cells and neutrophils (Figure 3a). Furthermore, mitotic activity was minimal, and most tumor cells showed low proliferative activity (MIB‐1 index). Immunohistochemical staining showed positivity for S‐100 protein and negativity for c‐kit, CD34, DOG1, and α‐SMA, leading to a definitive diagnosis of gastric schwannoma (Figure 3b–d).

Although the anemia improved with iron supplementation, surgical resection was recommended because of the symptomatic nature and large size of the lesion. However, the patient declined surgical treatment.

Eradication therapy for *H. pylori* infection was subsequently performed using vonoprazan fumarate, amoxicillin, and clarithromycin, and successful eradication was confirmed by a urea breath test. After the urea breath test, vonoprazan was resumed and continued until the follow‐up EGD and thereafter.

**FIGURE 4:**
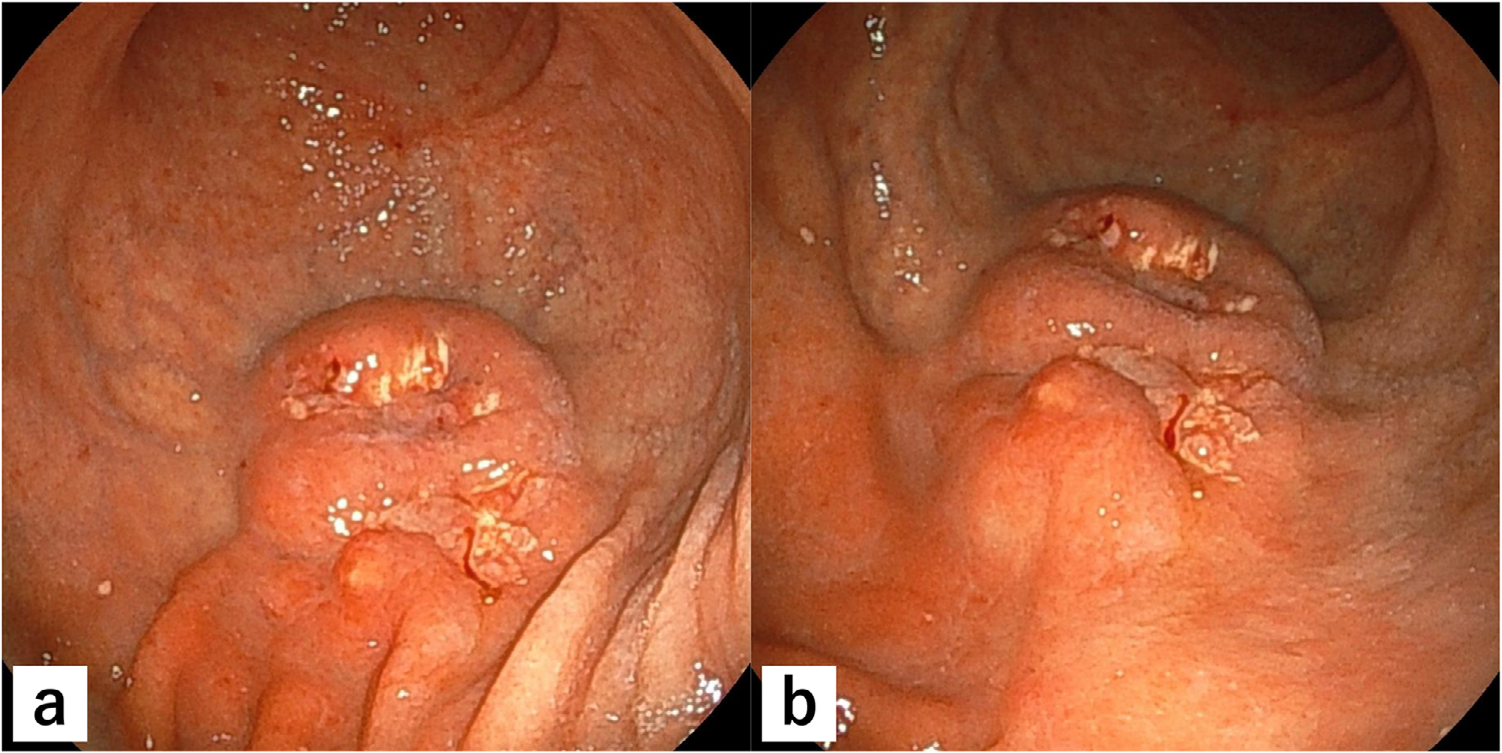
Follow‐up endoscopic findings. (a, b) Esophagogastroduodenoscopy performed 6 months after eradication showed healing of the surface ulceration and a change in the endoscopic appearance.

Follow‐up EGD performed 6 months after eradication showed healing of the ulcerative lesion and an apparent endoscopic decrease in the size of the tumor (Figure 4a,b). Thereafter, the patient remained asymptomatic, with no recurrence of anemia or dyspnea.

## 3 Discussion

Gastric schwannomas are rare mesenchymal tumors that are generally benign and slow‐growing. Although most cases are asymptomatic, larger lesions may present with ulceration, bleeding, or anemia due to mucosal disruption. However, gastric schwannomas with ulcer formation and clinically significant bleeding are uncommon, and such presentations may lead to diagnostic difficulty by mimicking other SELs, especially gastrointestinal stromal tumors [3]. To our knowledge, although ulcerated gastric schwannomas have been reported [6], there are very few reports describing an association with *H. pylori* infection, and no reports have described an apparent endoscopic decrease in the size of the tumor following eradication therapy.

In this case, the tumor measured more than 5 cm and was associated with ulceration and symptomatic anemia. Voltaggio et al. have stated that, although gastric schwannomas generally have a benign clinical course, complete excision and follow‐up would probably be warranted whenever reasonably feasible [3]. Accordingly, surgical resection was considered appropriate; however, conservative management was chosen at the patient's request.

A notable finding in this case was the healing of the ulcerative lesion and an apparent endoscopic decrease in the size of the tumor following eradication of *H. pylori*. *H. pylori* infection induces chronic active gastritis characterized by inflammatory cell infiltration and mucosal edema, which can lead to ulcer formation through mucosal injury [4, 5]. In contrast, ulceration in SELs is generally attributed to pressure‐induced ischemia and mechanical irritation of the overlying mucosa [7]. In the present case, these two mechanisms may have overlapped, resulting in exaggerated mucosal inflammation and an enlarged endoscopic appearance of the tumor. Improvement in local inflammatory and ulcer‐related changes after treatment, potentially resulting from both successful *H. pylori* eradication and continued acid suppression, may have contributed to ulcer healing and the apparent endoscopic change. These findings suggest that inflammatory or ulcer‐related changes associated with *H. pylori* infection may influence the endoscopic appearance of gastric SELs. Nevertheless, the apparent endoscopic decrease observed in this case cannot be attributed solely to successful *H. pylori* eradication. Other possible contributing factors include prolonged acid suppression with vonoprazan, spontaneous ulcer healing over time, resolution of mucosal edema, and technical differences between serial endoscopic examinations, such as the degree of insufflation, viewing angle, and observation distance. Because follow‐up EUS or computed tomography was not performed, the relative contribution of these factors cannot be determined, and true tumor regression cannot be confirmed. These considerations also represent important limitations of the present case, as objective measurements of tumor size and histopathological evidence of improvement in inflammation were unavailable, and long‐term follow‐up was limited. Further accumulation of similar cases is warranted to clarify this mechanism.

## Author Contributions

**Conceptualization**: Takahito Awatsu. **Methodology**: Takahito Awatsu. **Validation**: Takahito Awatsu, Daisuke Asaoka, Hirofumi Fukushima, Daishi Kabemura, Yusuke Nomoto, Osamu Nomura, Kazunori Kajino, Mariko Hojo, and Akihito Nagahara. **Investigation**: Takahito Awatsu, Mariko Hojo, and Kazunori Kajino. **Data curation**: Takahito Awatsu. **Writing – original draft preparation**: Takahito Awatsu. **Writing – review and editing**: Hirofumi Fukushima and Mariko Hojo. **Visualization**: Takahito Awatsu. **Supervision**: Akihito Nagahara.

## Funding

The authors have nothing to report.

## Conflicts of Interest

The authors declare no conflicts of interest.

## Data Availability

The data that support the findings of this study are available on request from the corresponding author. The data are not publicly available due to privacy or ethical restrictions.
